# Global multi‐specialty clinician perspectives on the implementation of Alzheimer's disease blood biomarkers

**DOI:** 10.1002/alz.70201

**Published:** 2025-05-22

**Authors:** Joanne Rodda, Lindsey A. Kuchenbecker, Wyllians V. Borelli, Mari L. DeMarco, Raphael M. Castilhos, Erin E. Cawston, Tinatin Chabrashvili, Melissa M. Budelier, Claudia Duran‐Aniotz, Chinedu Udeh‐Momoh, Leyla Akman‐Anderson, Michelle M. Mielke, Ana C. Pereira, Alicia Algeciras‐Schimnich, Ashvini Keshavan

**Affiliations:** ^1^ Kent and Medway Medical School Canterbury UK; ^2^ Kent and Medway NHS and Social Care Partnership Trust Maidstone UK; ^3^ Department of Neuroscience Mayo Clinic Jacksonville Florida USA; ^4^ Center for Clinical and Translational Science, Mayo Clinic Rochester Minnesota USA; ^5^ Graduate Program in Biological Sciences: Pharmacology and Therapeutics (PPGFT), UFRGS, Rua Ramiro Barcelos Porto Alegre Brazil; ^6^ Graduate Program in Biological Sciences: Biochemistry (PPGBioq), UFRGS, Rua Ramiro Barcelos Porto Alegre Brazil; ^7^ Department of Morphological Sciences UFRGS, Rua Sarmento Leite Porto Alegre Brazil; ^8^ Memory Center at the Hospital Moinhos de Vento, Rua Cel Aparício Borges Porto Alegre Brazil; ^9^ Department of Pathology and Laboratory Medicine St. Paul's Hospital, Providence Health Care Vancouver British Columbia Canada; ^10^ Department of Pathology and Laboratory Medicine University of British Columbia Vancouver British Columbia Canada; ^11^ Neurology Service, Hospital de Clínicas de Porto Alegre Porto Alegre Brazil; ^12^ Department of Pharmacology and Clinical Pharmacology and the Centre for Brain Research The University of Auckland Grafton Auckland New Zealand; ^13^ SUNY Upstate Medical University Syracuse New York USA; ^14^ TriCore Reference Laboratories Albuquerque New Mexico USA; ^15^ Latin American Institute for Brain Health (BrainLat) Universidad Adolfo Ibáñez Peñalolén Chile; ^16^ Department of Epidemiology and Prevention Wake Forest University School of Medicine Winston‐Salem North Carolina USA; ^17^ Brain and Mind Institute, Aga Khan University Nairobi Kenya; ^18^ NeuroVision Imaging, Inc., Garden Highway Sacramento California USA; ^19^ Department of Neurology Icahn School of Medicine at Mount Sinai New York New York USA; ^20^ Nash Family Department of Neuroscience Friedman Brain Institute, Icahn School of Medicine at Mount Sinai New York New York USA; ^21^ Department of Laboratory Medicine and Pathology Mayo Clinic Rochester Minnesota USA; ^22^ Dementia Research Centre, UCL Queen Square Institute of Neurology National Hospital for Neurology and Neurosurgery London UK

**Keywords:** Alzheimer's disease, biomarker, blood biomarkers, clinical implementation, cognitive impairment, diagnosis, primary care, secondary care

## Abstract

**INTRODUCTION:**

Clinicians’ views on the clinical readiness of Alzheimer's disease (AD) blood biomarkers (BBMs) are not well understood.

**METHODS:**

The Alzheimer's Association International Society to Advance Alzheimer's Research and Treatment Biofluid‐Based Biomarkers Professional Interest Area conducted a survey to elicit clinician opinions on AD BBM implementation, including contexts of use, assay selection, reporting, and result interpretation.

**RESULTS:**

Clinician respondents (*n* = 212) practiced in Europe (56%), North America (24%), the Caribbean and Central/South America (11%), and other continents (9%). Most respondents were medical doctors (80%) practicing in secondary or tertiary care (88%). For 56%, cerebrospinal fluid AD biomarkers or amyloid positron emission tomography were accessible, but 48% agreed and 52% disagreed with the implementation of AD BBMs in any clinical context. Respondents emphasized the need for data from diverse populations and educational resources to support test interpretation.

**DISCUSSION:**

Surveyed clinicians generally agreed with published appropriate use recommendations but were divided on AD BBM readiness for clinical use.

**Highlights:**

- A survey of clinicians was conducted regarding clinical readiness of Alzheimer's disease (AD) blood biomarkers (BBMs).
- Views were split on AD BBM clinical readiness: 48% agreed, 52% disagreed.
- Most responders supported AD BBM use for treatment decisions.
- Most responders opposed AD BBM testing in asymptomatic individuals.
- Test performance data and educational materials to aid interpretation were of high importance.

## 1 BACKGROUND

There is an increasing number of people living with Alzheimer's disease (AD) and related dementias (ADRD) globally.^1^ The landscape of AD has rapidly evolved with the advent of amyloid‐targeting disease‐modifying treatments (DMTs) for AD and intensified efforts to advance diagnostic tools. Eligibility for these treatments is predicated upon confirmation of amyloid beta (Aβ) pathology, which currently relies on positron emission tomography (PET) scans or cerebrospinal fluid (CSF) analysis. Regardless of eligibility for amyloid‐targeting therapies, many people with AD remain undiagnosed, experience long waits to diagnosis, or are misdiagnosed, while very few access biomarker testing.

AD blood biomarkers (BBMs) have emerged as easily accessible, low‐cost, scalable, and non‐invasive diagnostic tools.^2^, ^3^ AD BBMs can be broadly categorized based on their target pathology, including plasma Aβ42/40,^4^, ^5^ phosphorylated tau epitopes (p‐tau),^6^ indicators of neuronal degeneration (e.g., neurofilament light chain),^7^, ^8^ and markers of neuroinflammation (e.g., glial fibrillary acidic protein).^9^ Plasma p‐tau217 has the highest diagnostic accuracy for detection of amyloid pathology compared to other AD BBMs.^10^, ^11^, ^12^, ^13^, ^14^ The advancement and availability of highly accurate BBMs resulted in the incorporation of BBMs into the Alzheimer's Association's 2024 revised criteria for AD diagnosis as part of an integrated biological and clinical diagnostic and staging scheme.^3^ Many unresolved questions remain regarding application of AD BBMs across diverse settings and populations. Studies to date have been conducted predominantly in specialized settings and in non‐Hispanic White populations who are less likely than the general population to experience health or socioeconomic disadvantage. This is a major limitation because there is evidence that BBM levels differ based on racial and ethnic differences and are influenced by some chronic conditions and medications.^15^ Furthermore, the accuracy of BBM testing varies according to the prevalence of AD pathology in the population being tested, such that BBM testing in a younger cohort (e.g., ages 50–60) with no cognitive symptoms has a much lower positive predictive value for AD pathology than in an older population (e.g., > age 75) with cognitive decline.^16^ Additional complexity arises from the time interval of many years between the earliest detectable presence of AD pathology and symptom onset.^3^

The Global CEO Initiative has developed and published recommendations for the use of BBMs to support AD diagnosis.^2^, ^16^ These recommendations outline acceptable levels of accuracy for triaging and confirmation of diagnosis in both primary and secondary care settings, highlighting the importance of training and interpretation guidelines to optimize diagnostic utility. These reports underscore the potential for wider use of BBMs than the 2022 Alzheimer's Association's Appropriate Use Recommendations,^17^ potentially reflecting the rapidly growing evidence on the performance and utility of BBMs. Knowledge gaps remain in the clinical contexts for AD BBM implementation, with most published work focusing on use in secondary care and some in primary care.^2^, ^16^, ^17^, ^18^ The potential use of AD BBMs for wider population screening raises concerns about the impact of high false positive rates, and the identification of pathology in asymptomatic individuals where no treatment is available and the prognostic value is not fully understood, with implications for medical insurance, driving, and stigma.^19^, ^20^ Recently published World Health Organization Preferred Product Characteristics limit the recommended use of AD BBMs to people with progressive cognitive decline.^21^

The Alzheimer's Association International Society to Advance Alzheimer's Research and Treatment (ISTAART) Biofluid‐Based Biomarkers Professional Interest (BBB PIA) group was established in 2013 as the use of biofluid biomarkers expanded. The group's objective is to provide an effective means of communication between research and industry leaders for the development and advancement of clinical and research applications of ADRD biofluid biomarkers. Understanding real‐world clinician perspectives on the use of BBMs is critical to achieving this objective. Therefore, the group undertook an international survey of clinicians with the aim of capturing insights on the clinical utility, barriers, and perceived optimal applications of BBMs in AD diagnosis within their respective practices.

## 2 METHODS

### 2.1 Formulation of survey questions

The Real World Translation Work Group of the BBB PIA held an initial scoping meeting in September 2023. After this meeting, the work group chairs (A.A.S. and A.K.) drafted survey questions according to the following key headings: demographics of respondents, contexts of use, assay selection, reporting, and result interpretation. After a review by work group members at a meeting in November 2023, the draft survey underwent further iterations through December 2023 on a shared online document before the questions were finalized by the work group chairs, approved by ISTAART administration, and uploaded to the Qualtrics online platform. The final survey questions are detailed in Table S1 in supporting information. No personal identifiable information was collected, so the work group members and ISTAART administration agreed that research ethics committee approval would not be required to conduct the survey and publish the results in this position paper.

**RESEARCH IN CONTEXT**

1. **Systematic review**: The authors evaluated the literature on Alzheimer's disease (AD) blood biomarkers (BBMs), with particular focus on consensus reports on appropriate use and suggested clinical implementation. The Alzheimer's Association International Society to Advance Alzheimer's Research and Treatment Biofluid‐Based Biomarkers Professional Interest Area created and distributed a survey to assess real‐world multi‐specialty clinician perspectives on the clinical implementation of AD BBM.
2. **Interpretation**: Survey responses indicated that clinicians across specialties agreed with published consensus papers regarding the use of AD BBMs. Opinions were divided concerning the readiness of AD BBMs for clinical use and in which clinical settings their use is most appropriate.
3. **Future directions**: The clinical implementation of AD BBMs may be optimized by transparent reporting of assay performance and interpretative guidance for test results. More data are needed from diverse clinical settings to evaluate AD BBM performance in diverse patient populations, as tests become more accessible.

### 2.2 Dissemination of survey

The target respondents for the survey were practicing clinicians who would be the likely end users of clinically implemented AD BBM tests, across the various clinician roles and specialties that contribute to diagnosing patients presenting with cognitive symptoms. The work group aimed to gather a wide geographical representation and include the views of ISTAART members and non‐members. The methods of dissemination included emails to ISTAART membership, to other professional organizations with which the work group members had contact, advertisement of the survey on social media platforms (LinkedIn and X), and sharing of the QR code for the survey on presentations being given by work group members. The survey opened for responses over an initial period of January 18 to March 18, 2024, after which the work group decided to gather further responses by having a second open period of May 24[Table alz70201-tbl-0001] September 4, 2024, to overlap with the 2024 Alzheimer's Association International Conference.

### 2.3 Statistical analysis

Figures were generated using R (version 4.3.0)^22^ using ggplot2.^23^ Percentages included in the tables and text represent the proportion of responses to an answer choice relative to the total number of responses received from that survey question. The statistical significance of group differences was determined by chi‐square tests.

## 3 RESULTS

### 3.1 Survey respondent characteristics

A total of 212 clinicians participated in the survey (Table 1). The majority worked in secondary (42%) and tertiary (46%) care, with 12% working in primary care settings. Neurology was the predominant area of clinical practice (42%), followed by psychiatry (26%), gerontology (20%), and primary care (9%). Most respondents were attending physicians or consultants (71%), with 9% identifying in specialist nursing roles, 9% as residents or fellows, and 9% in other roles, including neuropsychologists and clinician researchers. Most respondents had > 10 years of clinical experience; 40% reported > 20 years and 32% reported 11 to 20 years of experience.

**TABLE 1 alz70201-tbl-0001:** Survey respondent characteristics.

Respondent characteristics (*n* = 212)	Response, *n* (%)
Care level	
Tertiary care	97 (46%)
Secondary care	90 (42%)
Primary care	25 (12%)
Main area of clinical practice in the field of ADRD	
Neurology	90 (42%)
Psychiatry	55 (26%)
Gerontology	43 (20%)
Primary care	19 (9%)
General internal medicine	5 (2)
Clinical role	
Medical doctor, attending physician, or consultant	150 (71%)
Medical doctor, resident, or fellow	20 (9%)
Nurse, consultant	20 (9%)
Other^a^	13 (6%)
Neuropsychologist	6 (3%)
Physician assistant/associate	3 (1%)
Level of clinical experience (number of years since qualifying)	
1–5 years	28 (13%)
6–10 years	33 (16%)
11–20 years	67 (32%)
> 20 years	84 (40%)
Location of current practice	
Europe	125 (59%)
North America	50 (24%)
Caribbean, Central America, or South America	24 (11%)
Australia, New Zealand, or Pacific Islands	5 (2%)
Asia	4 (2%)
Africa	2 (1%)
Middle East	2 (1%)
Currently active in AD biomarker research	
Yes	88 (42%)
No	124 (58%)
Able to request CSF AD biomarkers or amyloid PET on a clinical basis	
Yes	118 (56%)
No	94 (44%)
Length of time using CSF AD biomarkers or amyloid PET on a clinical basis	
< 1 year	16 (14%)
1–5 years	47 (40%)
6–10 years	26 (22%)
11–20 years	27 (23%)
> 20 years	2 (2%)

Abbreviations: AD, Alzheimer's disease; ADRD, Alzheimer's disease and related dementias; CSF, cerebrospinal fluid; PET, positron emission tomography.

^a^ Researcher (5); Clinical psychologist; Clinical neurologist; Industry, Neurochemist; Pharmacist; Occupational therapist; retired neurologist. One respondent who selected “Other” did not provide additional information on their clinical role.

**FIGURE 1 alz70201-fig-0001:**
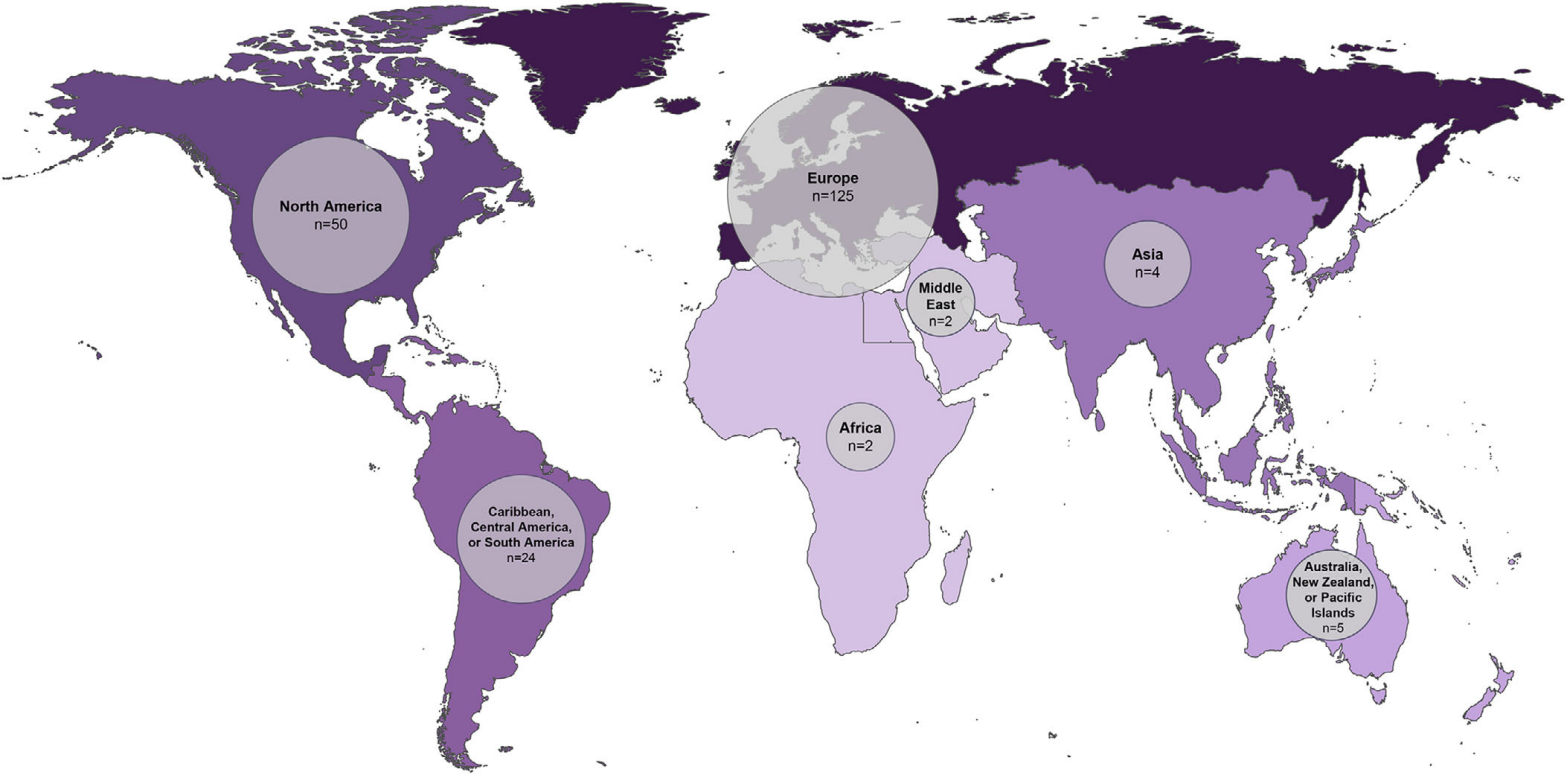
Geographic location of survey respondents: The location where survey respondents practice is depicted on a world map. Lighter shading indicates regions in which fewer respondents practice, and darker shading indicates regions in which a greater number of respondents practice. Bubble size describes the size of each regional subset in relation to the total cohort size.

The majority of respondents were based in Europe (59%); followed by North America (24%); the Caribbean, Central America, or South America (11%); Australia, New Zealand, or Pacific Islands (2%); Asia (2%); Africa (1%); and the Middle East (1%; Figure 1). Forty‐two percent of respondents reported that they were actively engaged in AD biomarker research. Just over half (56%) had the ability to request CSF AD biomarkers or amyloid PET scans in clinical practice. Fifty‐four percent of respondents with access to these tools reported using them for ≤ 5 years. Respondents in North America were most likely to have access to current gold standard biomarkers (84%), while these were available to fewer respondents from other areas, including the Caribbean, Central America, and South America (41.7%) and Europe (48%).

### 3.2 Use of blood biomarkers in clinical practice today

Opinions regarding the readiness of AD BBMs for use in clinical practice were divided, with 48% agreeing and 52% disagreeing with their implementation in any clinical context. The highest level of agreement regarding readiness for practice was in North America (62%), while 45% of respondents from Europe supported their use. The level of agreement for respondents from all other regions (Caribbean, Central America, South America, Africa, Middle East, Asia, Australia, New Zealand, Pacific Islands) was 38%. Primary care physicians were most likely to agree with AD BBM readiness for clinical practice (63.2%), followed by neurologists (52.9%), gerontologists (42%), and psychiatrists (36.4%). Of respondents who reported having access to current gold standard AD biomarkers, 52.1% agreed that AD BBMs were ready for use in clinical practice, compared to 41.9% of respondents who did not have access to current gold standard AD biomarkers (*p* = 0.14). Of those currently involved in AD BBM research, 50.6% answered that they were ready for use in clinical practice, while 45.5% not involved in AD BBM research answered yes to this question (*p* = 0.47). Additional survey responses regarding the readiness of AD biomarkers for clinical use and views on context of use stratified by respondent access to current gold standard AD biomarkers and engagement in AD biomarker research are shown in Table S2 and Table S3, respectively, in supporting information.[Fig alz70201-fig-0001]

Among those who supported implementation, the majority (89%) suggested that neurologists should be able to request these tests, while there was less support for access by other specialties, namely psychiatry (55%), gerontology (53%), and primary care (24%). When considering responses by clinical specialty, almost all respondents supported the use of AD BBMs by their own specialty and by neurologists, but less so by clinicians from other specialties (Table S4 in supporting information).

Respondents who felt that AD BBMs were not ready for clinical practice were asked what would increase their confidence in implementation (Table 2). The suggestions most strongly supported were: more data on biomarker performance within their patient population (73%); educational resources on test performance, validation, and interpretation (67%); and improved tools for health‐care professionals to interpret biomarker results (58%). Access to better patient counseling materials and multiple blood biomarker options were also highlighted by 48% and 26% of respondents, respectively.

**TABLE 2:** Views on readiness of AD blood biomarkers for use in clinical practice.

Survey questions	Response, *n* (%)
Are blood‐based AD biomarkers ready to be implemented in clinical practice, in any context of use?	
No	110 (52%)
Yes	100 (48%)
If yes, which specialties should be requesting these tests?	
Neurology	84 (89%)
Psychiatry	52 (55%)
Gerontology	50 (53%)
Primary care	23 (24%)
General internal medicine	20 (19%)
If no, what would increase your confidence in implementing blood‐based biomarkers in your practice?	
More data on performance of blood‐based biomarkers in research in my patient population	73 (20%)
Education on test performance, validation, and interpretation	67 (18%)
Tools to support interpretation of biomarker results by health‐care professionals	58 (16%)
Access to better material for patient counseling about blood‐based AD biomarkers	48 (48%)
Access to disease‐modifying therapies	36 (36%)
Increased access to gold standard biomarkers to confirm results of blood‐based biomarkers	26 (26%)
Increased access to multiple blood‐based biomarkers	26 (26%)
Other	13 (13%)

Abbreviation: AD, Alzheimer's disease.

**FIGURE 2 alz70201-fig-0002:**
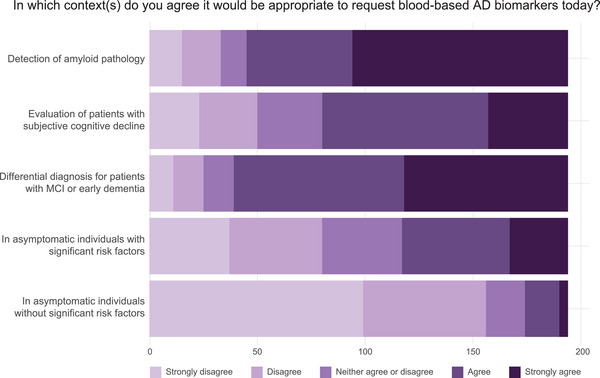
Survey responses regarding blood‐based AD biomarker context of use based on current knowledge. Bar plots display the proportion of survey respondents who agreed or disagreed with contexts of AD blood‐based biomarker use. Lighter shading indicates disagreement while darker shading indicates agreement. AD, Alzheimer's disease; MCI, mild cognitive impairment.

Most respondents strongly disagreed (51%) or disagreed (29%) with the use of AD BBMs in asymptomatic individuals without significant risk factors other than age; only 2% strongly agreed with their use in this context (Figure 2; Table S5 in supporting information). However, for asymptomatic individuals with significant risk factors (e.g., a significant family history or known high‐risk apolipoprotein E [*APOE*] genotype), 40% of respondents agreed or strongly agreed that testing would be appropriate.

Regarding symptomatic individuals, 59% of respondents agreed or strongly agreed with using BBMs in the evaluation of patients with subjective cognitive decline (SCD), while 80% supported their use in the differential diagnosis of mild cognitive impairment (MCI) or early dementia; 77% supported the use of BBMs for detecting amyloid pathology to guide treatment decisions involving disease‐modifying therapies.

### 3.3 Factors influencing blood biomarker test selection

**FIGURE 3 alz70201-fig-0003:**
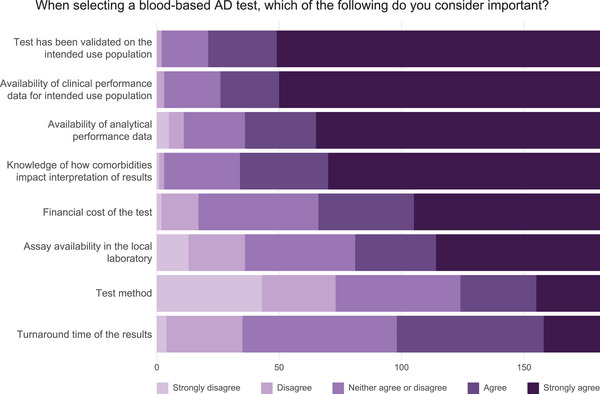
Factors influencing blood‐based biomarker test selection. Bar plots display the proportion of survey respondents who considered the importance of specific factors in selecting a blood‐based AD test. Lighter shading describes factors that are not considered to be important while darker shading indicates factors that respondents consider more important. AD, Alzheimer's disease.

The factors most frequently identified as fairly or very important when selecting a BBM test were validation in the population in which the test would be used (88%), availability of clinical performance data (85%), knowledge of how comorbidities might affect interpretation of results (81%), and availability of analytical performance data (80%; Figure 3; Table S6 in supporting information). Those less frequently rated as fairly or very important were financial cost (64%), assay availability in a local laboratory (55%), turnaround time for results (46%), and test method (31%).

### 3.4 Considerations when receiving biomarker test results

**FIGURE 4 alz70201-fig-0004:**
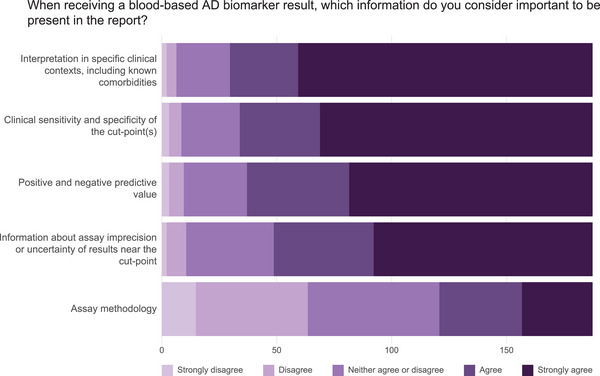
Considerations when reviewing biomarker test results. Bar plots display the proportion of survey respondents who considered the importance of specific factors in reviewing AD blood biomarker results. Lighter shading describes factors that are not considered to be important while darker shading indicates factors that respondents consider more important. AD, Alzheimer's disease.

Four of the five suggested factors for consideration when receiving a BBM result were rated as fairly or very important by 80% to 84% of respondents (Figure 4; Table S7 in supporting information). These factors are related to information on how to interpret the results (including sensitivity and specificity) in specific clinical contexts, information regarding assay imprecision or diagnostic uncertainty, and the positive and negative predictive value of the test. The assay methodology being used was perceived as the least important, with only 35% rating this as fairly or very important.

### 3.5 Pre‐test counseling and education for patients

**FIGURE 5 alz70201-fig-0005:**
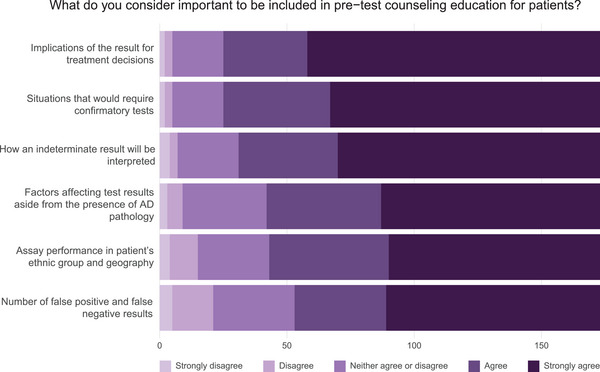
Pre‐test counseling and education for patients. Bar plots display the proportion of survey respondents who considered the importance of specific factors to be included in pre‐test counseling education for patients to undergo AD blood biomarker testing. Lighter shading describes factors that are not considered to be important while darker shading indicates factors that respondents consider more important. AD, Alzheimer's disease.

All of the items listed in the survey for possible inclusion in pre‐test counseling and education for patients were identified as fairly or very important by 70% to 85% of respondents (Figure 5; Table S8 in supporting information). Those most frequently identified as very important were the implications of the result for treatment decisions (66%), situations in which there will be a need for confirmatory tests (61%), and how an indeterminate result will be handled and interpreted (60%).

## 4 DISCUSSION

In this study, we present the findings of the first international survey of clinicians’ views regarding the use of AD BBMs in clinical practice. Responses from 212 clinicians across a range of specialties were captured, primarily from secondary and tertiary care settings. Respondents were based across six continents, with the majority from Europe and North America. Just under half of the respondents were involved in BBM research.[Fig alz70201-fig-0002], [Fig alz70201-fig-0003], [Fig alz70201-fig-0004], [Fig alz70201-fig-0005]

There was strongest concordance in views regarding the use of BBM in asymptomatic individuals (80% disagreed), use in MCI and early dementia (80% agreed), and for detection of amyloid pathology for treatment decisions with DMTs (77% agreed). Discordance was greatest for the key question of whether AD BBMs are ready for use in clinical practice (48% agreed, 52% disagreed) and use among patients with SCD (59% agree/strongly agree, 26% disagree/strongly disagree). Agreement with readiness of AD BBMs was greater in North America (62%) than in Europe (45%) or in other regions (38%). While clinicians with access to current gold standard AD biomarkers were more likely to support AD BBM use in clinical practice than those without access, involvement in AD BBM research appeared to be less of a factor in influencing opinions on readiness of AD BBM implementation.

Most respondents who supported the use of BBMs in current clinical practice agreed that neurologists should have access to testing, while there was much less support for their use by other professional groups. For those who did not support the use of BBMs in current clinical practice, education and access to population‐specific performance data were most frequently identified as factors that would increase confidence in BBM use.

The key priorities identified by respondents with respect to AD BBM test selection were the availability of analytic and clinical performance data, validation in the relevant population, and knowledge of the impact of comorbidities. There was general agreement that pre‐test counseling and education should include the implications of the result for treatment decisions, the possible need for additional confirmatory tests, handling of an indeterminate result, the impact of patient characteristics and demographics on the interpretation of results, and the possibility of false positives or negative results.

### 4.1 Clinician perspective

The most striking finding from the clinician's perspective is the division among respondents regarding the readiness of BBMs for use in clinical practice, with an almost even split of opinion. The concerns of those not supporting current clinical use of BBMs echoed those of recent consensus publications related to BBM implementation, namely the need for data from more diverse populations and for clinician education to ensure appropriate use and interpretation.^16^, ^18^

Also, in keeping with consensus publications, few respondents believed that AD BBM testing was appropriate in asymptomatic individuals without specific risk factors. This raises legal and ethical concerns about the possible consequences of direct‐to‐consumer BBM tests,^24^ both for individuals and health‐care systems. Similar concerns remain unresolved in direct‐to‐consumer genetic testing.^25^, ^26^ The use of BBMs in individuals with SCD was more accepted, perhaps reflecting the development of the concept of SCD as a category within the AD continuum,^27^ despite the most recent consensus on AD diagnosis not recommending the use of biomarkers in this group of individuals.^3^ Even among asymptomatic individuals, the presence of a risk *APOE* genotype and a family history of dementia leads to more divided responses. Overall, these findings suggest that many respondents consider it appropriate to use BBM testing in individuals without objective cognitive deficits but with risk factors for future cognitive decline.

A key barrier to accurate AD diagnosis is the limited availability of specialist services.^18^ BBMs offer the advantages of low cost and high scalability, yet overall, only approximately half of respondents who agreed that AD BBMs were ready for clinical implementation supported their use by psychiatrists or gerontologists, and fewer by primary care, while the vast majority endorsed their use by neurologists. Given that a small minority of dementia diagnoses are made within neurological services,^28^, ^29^ such restrictions would reduce the potential impact of BBMs. Broader clinician education and data on BBM use in diverse populations may increase confidence in their use across different specialties. Furthermore, the finding that clinicians generally supported the use of AD BBMs by their own specialty demonstrates a clear need for cross‐specialty collaboration and improved awareness of different ways of working.

### 4.2 Challenges and opportunities in low and middle income countries

The low level of representation of clinicians from low and middle income countries (LMICs) in the survey (15%) highlights a broader issue and reflects the significant paucity of data and publications on BBMs from LMICs. It is critical that LMIC researchers and institutions are proactively engaged in the development, validation, and clinical translation of research on BBMs in AD to address this gap and promote equitable progress in the field.

The clinical implementation of BBMs provides an exciting opportunity for advancing AD diagnosis in LMICs. However, real‐world effectiveness, cost efficiency, and acceptance remain underexplored in resource‐limited settings. Limited financial resources hinder the development and validation of these tools in LMICs, which is essential for clinical implementation.^20^, ^30^, ^31^ In addition, most BBM studies focus on homogeneous populations in high‐income countries (HICs), highlighting the urgent need for data from large international studies capturing underrepresented groups with different rates of comorbidities, and varied socio‐economic, ethnic, and genetic backgrounds.^32^, ^33^, ^34^ Alongside this, cultural perceptions of AD BBMs must be investigated, requiring a deeper discussion with policy makers and other stakeholders.

Scarce infrastructure and the need for capacity building in LMICs would require financial investment from multiple sources, upskilling of the workforce, and engagement by companies, especially in remote areas, to implement widespread BBM use. Pragmatic approaches, including the use of dried blood spots^35^ and task‐shifting models that enable community health workers to collect samples,^36^ could enhance implementation.

Currently, very few people in LMICs have access to AD biomarkers, so BBMs may present their only opportunity to detect AD pathology. Ultimately, the study of BBMs in diverse environments will enable a deeper understanding of how social determinants and life histories impact biomarker profiles and improve global brain health and dementia research models.^37^, ^38^

### 4.3 Laboratory medicine perspective

Clinical laboratories will play a critical role in supporting clinicians by providing essential information for selecting and interpreting AD BBM tests. Survey findings suggest that many clinicians using BBMs will have limited experience with them. Therefore, laboratories must provide clear and accessible data to aid test selection, patient counseling, and integration of results into clinical decision making.

Validation data are paramount in clinicians’ test selection; analytical and clinical performance metrics, along with factors influencing these metrics, such as patient comorbidities and disease prevalence in specific settings, ranked highly in importance in the survey. Laboratories should report comprehensive performance metrics and clearly describe the cohorts used to derive them, including demographic and clinical characteristics.

Survey responses suggest that laboratories should enhance test catalogues by including detailed validation data and interpretive support. Laboratories should transparently disclose the rigor of the validation and associated data, so that ordering providers can make informed decisions. Respondents also prioritized access to detailed performance metrics and modifiers of these metrics for test result reporting. Laboratories can reinforce appropriate use of BBM testing by linking results to validation data and interpretive guidance. Laboratories should avoid references to biomarker platforms/data not directly offered by their laboratory, which could lead to misinterpretation or inappropriate use.

Less critical factors in test selection included test method, local availability, turnaround time, and cost. The lower priority of these factors reflects clinicians’ emphasis on performance and interpretive support. While cost was not a major consideration in this survey of clinicians from primarily HICs, it may well be prioritized differently in lower resource settings or among other stakeholders, such as patients, laboratories, and insurers.

Future research into clinician perspectives on AD BBM use could include gathering perspectives of a larger sample of global clinicians, and more qualitative methods such as interviews and/or focus groups. Key questions to be addressed include the nature of validation of AD BBMs in specific populations that would provide clinicians with sufficient confidence to use them in clinical practice (e.g., numbers of participants included, types of population studied). A further question to explore is the rationale for the responses of participants who agreed that use of AD BBMs in asymptomatic individuals would be appropriate, given that this is not recommended by current published guidelines. Finally, access to educational resources was identified as a barrier to the use of AD BBMs in clinical practice; therefore, a deeper understanding of the types of educational resources preferred by different groups of clinicians would be valuable.

### 4.4 Limitations

Despite efforts to engage clinicians globally through ISTAART and encourage information sharing among colleagues, only a small number of respondents were from LMICs, and the majority were from North America and Europe. Similarly, most respondents were experienced clinicians working in specialist environments with access to CSF biomarkers, which is not the case for most clinicians responsible for dementia diagnosis. Furthermore, the sample size overall represents only a small proportion of clinicians globally who may use BBMs in their practice. Although these factors limit generalizability, the survey has captured views from clinicians across the globe regarding a wide range of issues related to the clinical implementation of BBMs and has improved our understanding of clinician‐related facilitators and challenges to their clinical implementation.

### 4.5 Conclusion

In conclusion, the findings of this study demonstrate that clinician views are in broad agreement with published consensus papers regarding the use of AD BBMs. Findings also emphasize the need for data from more diverse populations, and education related to test performance, validation, and interpretation. The study also highlights divided opinion, even among specialists, regarding the readiness of AD BBMs for clinical use, and the appropriateness of various clinical settings. To optimize BBM implementation, clinical laboratories must prioritize transparent reporting of assay performance and interpretive guidance. The survey primarily captured specialist views from HICs; our future research will explore opinions in more diverse clinical settings, tracking evolving views as BBM testing becomes more accessible.

## CONFLICT OF INTEREST STATEMENT

W.V.B. has served on the scientific advisory board of Masima, and he is also a co‐founder and a minority shareholder at Masima. M.L.D. participates on advisory boards for Eisai and Roche, is a co‐chair for the Academy of Diagnostics and Laboratory Medicine guidance document on AD biomarkers, and has received in‐kind support for laboratory materials from Meso Scale Discovery, Fujirebio, and Roche. R.M.C. has participated on an advisory board for Biogen and receives an Alzheimer's Association research grant. M.M.B. reports advisory board participation for Roche Diagnostics and Greiner Bio‐One; travel support and lecture fees from Roche Diagnostics and Bio‐Rad; licensing income from technology licensed by Washington University to C2N Diagnostics; and is a co‐inventor on the following patents related to Alzheimer's disease testing: 018941/US, PCT/US2022/015998. Although not directly funding this work, C.U.M. additionally receives funding support from The Alzheimer's Association (SAGA23‐1141999), Wellcome Leap and Temasek Trust Dynamic Resilience Award, National Institutes of Health (NIH; RO1‐AG074562), Office for Veterans’ Affairs UK Defense and Security Accelerator (DASA) Fund (G2‐SCH‐2022‐11‐12245), Global Brain Health Institute (UFRA‐424|CA‐0241758), Davos Alzheimer's Collaborative Global Cohorts Fund, and RoseTrees Foundation (Seedcorn2021∖100220). L.A.A. is an inventor on a patent pending (EP‐22‐772‐019.0). M.M.M. has served on scientific advisory boards and/or has consulted for Acadia, Althira, Biogen, Eisai, Lilly, Merck, Novo Nordisk, and Roche; received speaking honoraria from Novo Nordisk, PeerView Institute, and Roche; and receives grant support from the Alzheimer's Association. A.C.P. has patents unrelated to this work licensed to Neurobiopharma, LLC; serves on the scientific advisory board of Sinaptica Therapeutics and has served as a consultant to Eisai and Quanterix; and receives grant support from the Alzheimer's Association. AAS has participated on advisory boards for Roche Diagnostics and Fujirebio Diagnostics, and has received speaker honoraria from Roche Diagnostics. A.K., J.R., L.A.K., E.E.C., and C.D.A. report no conflicts of interest. Author disclosures are available in the supporting information.

## CONSENT STATEMENT

It was not necessary for human subjects to provide informed consent for this study.

## Supporting information



Supporting Information

Supporting Information
