# Supplementary material for: Global multi‐specialty clinician perspectives on the implementation of Alzheimer's disease blood biomarkers
Source: Alzheimers Dement. 2025 May 22;21(5):e70201. doi: 10.1002/alz.70201 (PMC12096314; doi:10.1002/alz.70201)
Supplement: Supplementary file 2 — Supporting Information [file ALZ-21-e70201-s002.docx]

**Supplementary Materials**

**Supplementary Table 1**: Complete survey questions and responses

| **Question** | **Response, n (%)** |
| --- | --- |
| **Q1 - In which setting do you work most frequently?** | |
| Primary Care | 25 (12%) |
| Secondary Care | 90 (42%) |
| Tertiary Care | 97 (46%) |
| **Q2 - What is your main area of clinical practice in the field of ADRD?** | |
| Primary Care | 19 (9%) |
| Neurology | 90 (42%) |
| Psychiatry | 55 (26%) |
| Gerontology | 43 (20%) |
| General Internal Medicine | 5 (2%) |
| **Q3 - What is your clinical role?** | |
| Medical doctor - attending physician/consultant | 150 (71%) |
| Medical doctor - resident/fellow | 20 (9%) |
| Nurse - consultant | 2 (1%) |
| Nurse - advanced practitioner | 14 (7%) |
| Physician assistant/associate | 3 (1%) |
| Other: Researcher; Neuropsychologist; Neuropsychologist; Pharmacist; Clinical neuropsychologist; Neuropsychologist; Clinical psychologist; Clinical researcher; Assistant research professor; Industry; Neurochemist; CNS; Community MH nurse; Clinical neurologist; Clinical nurse specialist; Occupational therapist; Retired neurologist; PhD candidate; Clinical lead nurse; Neuropsychologist; Postdoctoral fellow; Neuropsychologist | 23 (11%) |
| **Q4 - What is your level of clinical experience (years since qualifying)?** | |
| 1-5 | 28 (13%) |
| 6-10 | 33 (16%) |
| 11-20 | 67 (32%) |
| >20 | 84 (40%) |
| **Q5 - Where do you currently practice?** | |
| North America | 50 (24%) |
| Caribbean, Central America, or South America | 24 (11%) |
| Europe | 125 (59%) |
| Africa | 2 (1%) |
| Middle East | 2 (1%) |
| Asia | 4 (2%) |
| Australia, New Zealand, or Pacific Islands | 5 (2%) |
| **Q6 - Are you currently active in AD biomarker research (published research on any AD biomarkers within the last 10 years)?** | |
| Yes | 88 (42%) |
| No | 124 (58%) |
| **Q7 - Do you currently have access to/are you able to request "gold standard" AD biomarkers (CSF or amyloid PET) for your patients on a clinical basis?** | |
| Yes | 118 (56%) |
| No | 94 (44%) |
| **Q8 - If yes, for how long have you been using and interpreting CSF AD biomarkers or amyloid PET for your patients on a clinical basis?** | |
| <1 year | 16 (14%) |
| 1-5 years | 47 (40%) |
| 6-10 years | 26 (22%) |
| 11-20 years | 27 (23%) |
| >20 years | 2 (2%) |
| **Q9 - In your opinion, are blood-based AD biomarkers ready to be implemented in clinical practice, in any context of use?** | |
| Yes | 100 (48%) |
| No | 110 (52%) |
| **Q10 - If yes, which specialties should be requesting these tests? Select all that apply** | |
| Primary Care | 23 (10%) |
| Neurology | 84 (37%) |
| Psychiatry | 52 (23%) |
| Gerontology | 50 (22%) |
| General Internal Medicine | 19 (8%) |
| **Q11 - If no, what would increase your confidence in implementing blood-based AD biomarkers in your practice? Select all that apply.** | |
| Education on test performance, validation, and interpretation | 67 (18%) |
| Increased access to gold standard biomarkers to confirm results of blood-based AD biomarkers | 47 (13%) |
| Increased access to multiple blood-based biomarkers | 26 (7%) |
| More data on performance of blood-based AD biomarkers in research in my patient population | 73 (20%) |
| Access to better material for patient counselling about blood-based AD biomarkers | 48 (13%) |
| Tools to support interpretation of biomarker results by healthcare professionals | 58 (16%) |
| Access to disease-modifying therapies | 36 (10%) |
| Other: create awareness; N/A; blood biomarkers are not ready for clinical use they need developing further; cost; greater sensitivity for detecting clinically relevant Alzheimer's Disease; they overlap so are impossible to interpret except at far + or far neg they take too long to return; clinical validity in asymptomatic patients; evidence that any intervention is useful for a patient group with positive biomarkers but no symptoms; better tests with clearer discrimination between disease and non-disease groups; more information on the interpretation of BBB in the case of chronic renal failure other comorbidities like previous stroke and heart disease; demonstration of performance in diverse populations, multiple underlying pathologies and atypical AD (e.g. language variant); all could be helpful; higher specificity | 13 (4%) |
| **Q12 - In which context(s) do you agree it would be appropriate to request blood-based AD biomarkers today?** | |
| **In asymptomatic individuals without significant risk factors beyond age** |  |
| Strongly disagree | 99 (51%) |
| Disagree | 57 (29%) |
| Neither agree or disagree | 18 (9%) |
| Agree | 16 (8%) |
| Strongly agree | 4 (2%) |
| **In asymptomatic individuals with significant risk factors (e.g. a significant family history, or known high risk APOE genotype) - as a susceptibility/risk biomarker** |  |
| Strongly disagree | 37 (19%) |
| Disagree | 43 (22%) |
| Neither agree or disagree | 37 (19%) |
| Agree | 50 (26%) |
| Strongly agree | 27 (14%) |
| **In evaluation of patients with subjective cognitive decline** |  |
| Strongly disagree | 23 (12%) |
| Disagree | 27 (14%) |
| Neither agree or disagree | 30 (15%) |
| Agree | 77 (40%) |
| Strongly agree | 37 (19%) |
| **In the differential diagnosis of patients presenting with mild cognitive impairment or early dementia** |  |
| Strongly disagree | 11 (6%) |
| Disagree | 14 (7%) |
| Neither agree or disagree | 14 (7%) |
| Agree | 79 (41%) |
| Strongly agree | 76 (39%) |
| **For detecting amyloid pathology for treatment decisions with disease modifying therapies** |  |
| Strongly disagree | 15 (8%) |
| Disagree | 18 (9%) |
| Neither agree or disagree | 12 (6%) |
| Agree | 49 (25%) |
| Strongly agree | 100 (52%) |
| **Q13 - Are blood-based AD biomarkers currently approved for clinical use and available for you to request for any patients in your practice?** | |
| Yes | 35 (18%) |
| No | 159 (82%) |
| **Q14 - If yes, how often have they changed patient management?** | |
| Almost always | 4 (12%) |
| Usually | 5 (15%) |
| About half the time | 9 (27%) |
| Seldom | 5 (15%) |
| Almost never | 10 (30%) |
| **Q15 - Are amyloid-targeting therapies for AD currently approved for clinical use and available to any patients in your practice, or expected to be available imminently (e.g. one year from now)?** | |
| Yes | 77 (40%) |
| No | 115 (60%) |
| **Q16 - When selecting a blood-based AD test, which of the following do you consider important? Rate each statement using one of the five choices below.** | |
| **Test method (i.e. immunoassay versus mass spectrometry)** |  |
| Not at all important | 43 (24%) |
| Slightly important | 30 (17%) |
| Important | 51 (28%) |
| Fairly important | 31 (17%) |
| Very important | 26 (14%) |
| **Assay availability in the local laboratory** |  |
| Not at all important | 13 (7%) |
| Slightly important | 23 (13%) |
| Important | 45 (25%) |
| Fairly important | 33 (18%) |
| Very important | 67 (37%) |
| **Analytical performance data are available for the test being ordered (e.g. precision, accuracy)** |  |
| Not at all important | 5 (3%) |
| Slightly important | 6 (3%) |
| Important | 25 (14%) |
| Fairly important | 29 (16%) |
| Very important | 116 (64%) |
| **Clinical performance data are available for the test being ordered and the intended use population (e.g. sensitivity and specificity in comparison to gold standard biomarkers)** |  |
| Not at all important | 0 (0%) |
| Slightly important | 3 (2%) |
| Important | 23 (13%) |
| Fairly important | 24 (13%) |
| Very important | 131 (72%) |
| **Test has been validated on the population that would be using the test (e.g. clinical status, diversity** |  |
| Not at all important | 0 (0%) |
| Slightly important | 2 (1%) |
| Important | 19 (10%) |
| Fairly important | 28 (15%) |
| Very important | 132 (73%) |
| **Turn-around time of the results** |  |
| Not at all important | 4 (2%) |
| Slightly important | 31 (17%) |
| Important | 63 (35%) |
| Fairly important | 60 (33%) |
| Very important | 23 (13%) |
| **Knowledge of how comorbidities (i.e. chronic kidney disease, obesity, other health conditions) might affect the interpretation of the test being ordered** |  |
| Not at all important | 1 (1%) |
| Slightly important | 2 (1%) |
| Important | 31 (17%) |
| Fairly important | 36 (20%) |
| Very important | 111 (61%) |
| **Financial cost of the test (to the health care system or to the patient)** |  |
| Not at all important | 2 (1%) |
| Slightly important | 15 (8%) |
| Important | 49 (27%) |
| Fairly important | 39 (22%) |
| Very important | 76 (42%) |
| **Q17 - When receiving a blood-based AD biomarker result, which information do you consider important to be present in the report? Rate each statement using one of the five choices below.** | |
| **Information about the assay imprecision or diagnostic uncertainty of results near the clinical decision limit cut-point** |  |
| Not at all important | 2 (1%) |
| Slightly important | 8 (5%) |
| Important | 36 (20%) |
| Fairly important | 41 (23%) |
| Very important | 90 (51%) |
| **Assay methodology being used** |  |
| Not at all important | 14 (8%) |
| Slightly important | 46 (26%) |
| Important | 54 (31%) |
| Fairly important | 34 (19%) |
| Very important | 29 (16%) |
| **Clinical sensitivity and specificity of the cut-point(s) used in the assay in specified contexts of use relevant for my setting** |  |
| Not at all important | 3 (2%) |
| Slightly important | 5 (3%) |
| Important | 24 (14%) |
| Fairly important | 33 (19%) |
| Very important | 112 (63%) |
| **Positive and negative predictive value of the test** |  |
| Not at all important | 3 (2%) |
| Slightly important | 6 (3%) |
| Important | 26 (15%) |
| Fairly important | 42 (24%) |
| Very important | 100 (56%) |
| **Information on how to interpret a result in specific clinical contexts, including known comorbidities** |  |
| Not at all important | 2 (1%) |
| Slightly important | 4 (2%) |
| Important | 22 (12%) |
| Fairly important | 28 (16%) |
| Very important | 121 (68%) |
| **Q18 - What do you consider important to be included in pre-test counselling education for patients? Rate each statement using one of the five choices below.** | |
| **Numbers of false positive and false negative results** |  |
| Not at all important | 4 (2%) |
| Slightly important | 11 (6%) |
| Important | 28 (16%) |
| Fairly important | 47 (27%) |
| Very important | 83 (48%) |
| **Factors that may affect the test results aside from the presence of AD pathology (demographics, comorbidities)** |  |
| Not at all important | 3 (2%) |
| Slightly important | 6 (3%) |
| Important | 33 (19%) |
| Fairly important | 45 (26%) |
| Very important | 86 (50%) |
| **Assay performance in patient's ethnic group and geography** |  |
| Not at all important | 5 (3%) |
| Slightly important | 16 (9%) |
| Important | 32 (18%) |
| Fairly important | 36 (21%) |
| Very important | 84 (49%) |
| **Implications of the result for treatment decisions** |  |
| Not at all important | 2 (1%) |
| Slightly important | 3 (2%) |
| Important | 20 (12%) |
| Fairly important | 33 (19%) |
| Very important | 115 (66%) |
| **How an indeterminate result will be handled/interpreted** |  |
| Not at all important | 4 (2%) |
| Slightly important | 3 (2%) |
| Important | 24 (14%) |
| Fairly important | 39 (23%) |
| Very important | 103 (60%) |
| **Situations in which there will be a need for confirmatory tests (CSF/amyloid PET)** |  |
| Not at all important | 2 (1%) |
| Slightly important | 3 (2%) |
| Important | 20 (12%) |
| Fairly important | 42 (24%) |
| Very important | 106 (61%) |

Abbreviations: AD = Alzheimer’s disease; *APOE* = apolipoprotein-E gene.

**Supplementary Table 2: Views on readiness of AD blood biomarkers for use in clinical practice and context of use stratified by respondent access to current gold standard AD biomarkers**

| **Survey questions** | **Response, n (%)** | |
| --- | --- | --- |
|  | Respondents who currently use gold-standard AD biomarkers | Respondents who do not currently use gold-standard AD biomarkers |
| Are blood-based AD biomarkers ready to be implemented in clinical practice, in any context of use?   - *No* - *Yes* | 56 (47.9%)  61 (52.1%) | 54 (58.1%)  39 (41.9%) |
| If yes, which specialties should be requesting these tests?   - *Neurology* - *Psychiatry* - *Gerontology* - *Primary care* - *General internal medicine* | 52 (91.2%)  24 (42.1%)  23 (40.4%)  10 (17.5%)  8 (14.0%) | 32 (86.5%)  28 (75.7%)  27 (73.0%)  13 (35.1%)  11 (29.7%) |
| If no, what would increase your confidence in implementing blood-based biomarkers in your practice?   - *More data on performance of blood-based biomarkers in research in my patient population* - *Education on test performance, validation and interpretation* - *Tools to support interpretation of biomarker results by healthcare professionals* - *Access to better material for patient counselling about blood-based AD biomarkers* - *Access to disease-modifying therapies* - *Increased access to gold standard biomarkers to confirm results of blood-based biomarkers* - *Increased access to multiple blood-based biomarkers* - *Other* | 38 (71.7%)  31 (58.5%)  27 (50.9%)  20 (37.7%)  14 (26.4%)  25 (47.2%)  10 (18.9%)  9 (17.0%) | 35 (74.5%)  36 (76.6%)  31 (66.0%)  28 (59.6%)  22 (46.8%)  22 (46.8%)  16 (34.0%)  4 (8.5%) |
| In asymptomatic individuals without significant risk factors beyond age   - *Strongly disagree* - *Disagree* - *Neither agree nor disagree* - *Agree* - *Strongly agree* | 64 (58.2%)  30 (27.3%  10 (9.1%)  3 (2.7%)  3 (2.7%) | 35 (41.7%)  27 (32.1%)  8 (9.5%)  13 (15.5%)  1 (1.2%) |
| In asymptomatic individuals with significant risk factors (e.g. a significant family history, or known high risk *APOE* genotype)   - *Strongly disagree* - *Disagree* - *Neither agree nor disagree* - *Agree* - *Strongly agree* | 28 (25.5%)  30 (27.3%)  18 (16.4%)  23 (20.9%)  11 (10.0%) | 9 (10.7%)  13 (15.5%)  19 (22.6%)  27 (23.1%)  16 (19.0%) |
| In evaluation of patients with subjective cognitive decline   - *Strongly disagree* - *Disagree* - *Neither agree nor disagree* - *Agree* - *Strongly agree* | 15 (13.6%)  15 (13.6%)  16 (14.5%)  42 (38.2%)  22 (20.0%) | 8 (9.5%)  12 (14.3%)  14 (16.7%)  35 (41.7%)  15 (17.9%) |
| In the differential diagnosis of patients presenting with mild cognitive impairment or early dementia   - *Strongly disagree* - *Disagree* - *Neither agree nor disagree* - *Agree* - *Strongly agree* | 9 (8.2%)  7 (6.4%)  8 (7.3%)  35 (31.8%  51 (46.4%) | 2 (2.4%)  7 (8.3%)  6 (7.1%)  44 (52.4%)  25 (29.8%) |
| For detecting amyloid pathology for treatment decisions with disease modifying therapies   - *Strongly disagree* - *Disagree* - *Neither agree nor disagree* - *Agree* - *Strongly agree* | 15 (7.7%)  18 (9.3%)  12 (6.2%)  49 (25.3%)  100 (51.5%) | 2 (2.4%)  8 (9.5%)  6 (7.1%)  25 (29.8%)  43 (51.2%) |

Abbreviations: AD = Alzheimer’s disease; *APOE* = apolipoprotein-E gene.

**Supplementary Table 3: Views on readiness of AD blood biomarkers for use in clinical practice and context of use stratified by respondent engagement in biomarker research**

| **Survey questions** | **Response, n (%)** | |
| --- | --- | --- |
|  | Respondents currently engaged in AD biomarker research | Respondents not currently engaged in AD biomarker research |
| Are blood-based AD biomarkers ready to be implemented in clinical practice, in any context of use?   - *No* - *Yes* | 43 (49.4%)  44 (50.6%) | 67 (54.5%)  56 (45.5%) |
| If yes, which specialties should be requesting these tests?   - *Neurology* - *Psychiatry* - *Gerontology* - *Primary care* - *General internal medicine* | 37 (86.0%)  15 (34.9%)  14 (32.6%)  9 (20.9%)  5 (11.6%) | 47 (92.2%)  37 (72.5%)  36 (70.6%)  14 (27.5%)  14 (27.5%) |
| If no, what would increase your confidence in implementing blood-based biomarkers in your practice?   - *More data on performance of blood-based biomarkers in research in my patient population* - *Education on test performance, validation and interpretation* - *Tools to support interpretation of biomarker results by healthcare professionals* - *Access to better material for patient counselling about blood-based AD biomarkers* - *Access to disease-modifying therapies* - *Increased access to gold standard biomarkers to confirm results of blood-based biomarkers* - *Increased access to multiple blood-based biomarkers* - *Other* | 30 (75.0%)  20 (50.0%)  19 (47.5%)  13 (32.5%)  5 (12.5%)  17 (42.5%)  8 (20.%)  5 (12.5%) | 43 (71.7%)  47 (78.3%)  39 (65.0%)  35 (58.3%)  31 (51.7%)  30 (50.0%)  18 (30.0%)  8 (13.3%) |
| In asymptomatic individuals without significant risk factors beyond age   - *Strongly disagree* - *Disagree* - *Neither agree nor disagree* - *Agree* - *Strongly agree* | 48 (57.8%)  24 (28.9%)  4 (4.8%)  4 (4.8%)  3 (3.6%) | 51 (45.9%)  33 (29.7%)  14 (12.6%)  12 (10.8%)  1 (0.9%) |
| In asymptomatic individuals with significant risk factors (e.g. a significant family history, or known high risk *APOE* genotype)   - *Strongly disagree* - *Disagree* - *Neither agree nor disagree* - *Agree* - *Strongly agree* | 25 (30.1%)  21 (25.3%)  13 (15.7%)  14 (16.9%)  10 (12.0%) | 12 (10.8%)  22 (19.8%)  24 (21.6%)  36 (32.4%)  17 (15.3%) |
| In evaluation of patients with subjective cognitive decline   - *Strongly disagree* - *Disagree* - *Neither agree nor disagree* - *Agree* - *Strongly agree* | 13 (15.7%)  14 (16.9%)  11 (13.3%)  27 (32.5%)  18 (21.7%) | 10 (9.0%)  13 (11.7%)  19 (17.1%)  50 (45.0%)  19 (17.1%) |
| In the differential diagnosis of patients presenting with mild cognitive impairment or early dementia   - *Strongly disagree* - *Disagree* - *Neither agree nor disagree* - *Agree* - *Strongly agree* | 6 (7.2%)  7 (8.4%)  9 (10.8%)  23 (27.7%)  38 (45.8%) | 5 (4.5%)  7 (6.3%)  5 (4.5%)  56 (50.5%)  38 (34.2%) |
| For detecting amyloid pathology for treatment decisions with disease modifying therapies   - *Strongly disagree* - *Disagree* - *Neither agree nor disagree* - *Agree* - *Strongly agree* | 9 (10.8%)  11 (13.3%)  5 (6.0%)  20 (24.1%)  38 (45.8%) | 6 (5.4%)  7 (6.3%)  7 (6.3%)  29 (26.1%)  62 (55.9%) |

Abbreviations: AD = Alzheimer’s disease; *APOE* = apolipoprotein-E gene.

**Supplementary Table 4:** Views on the implementation of blood AD biomarkers in specific clinical specialties

| **Respondent specialty** | **N** | **Agree with implementation in clinical specialty*, n (%)** | | | |
| --- | --- | --- | --- | --- | --- |
|  |  | **Neurology** | **Psychiatry** | **Gerontology** | **Primary care or general internal medicine** |
| Neurology | 46 | 44 (96%) | 18 (39%) | 16 (35%) | 6 (13%) |
| Psychiatry | 20 | 18 (90%) | 17 (85%) | 14 (70%) | 8 (40%) |
| Gerontology | 17 | 15 (88%) | 12 (71%) | 17 (100%) | 7 (41%) |
| Primary care and general internal medicine | 11 | 7 (64%) | 5 (45%) | 3 (27%) | 10 (91%) |

*Counts and proportions represent respondents who answered “Yes” to survey question “In your opinion, are blood AD biomarkers ready to be implemented in clinical practice, in any context of use?” and responded to survey question “If yes, which specialties should be requesting these tests? Select all that apply”.

**Supplementary Table 5:** Survey responses regarding blood AD biomarker context of use based on current knowledge

| **Survey questions** | **Response, n (%)** |
| --- | --- |
| In asymptomatic individuals without significant risk factors beyond age   - *Strongly disagree* - *Disagree* - *Neither agree nor disagree* - *Agree* - *Strongly agree* | 99 (51%)  57 (29%)  18 (9%)  16 (8%)  4 (2%) |
| In asymptomatic individuals with significant risk factors (e.g. a significant family history, or known high risk *APOE* genotype)   - *Strongly disagree* - *Disagree* - *Neither agree nor disagree* - *Agree* - *Strongly agree* | 37 (19%)  43 (22%)  37 (19%)  50 (26%)  27 (14%) |
| In evaluation of patients with subjective cognitive decline   - *Strongly disagree* - *Disagree* - *Neither agree nor disagree* - *Agree* - *Strongly agree* | 23 (12%)  27 (14%)  30 (15%)  77 (40%)  37 (19%) |
| In the differential diagnosis of patients presenting with mild cognitive impairment or early dementia   - *Strongly disagree* - *Disagree* - *Neither agree nor disagree* - *Agree* - *Strongly agree* | 11 (6%)  14 (7%)  14 (7%)  79 (41%)  76 (39%) |
| For detecting amyloid pathology for treatment decisions with disease modifying therapies   - *Strongly disagree* - *Disagree* - *Neither agree nor disagree* - *Agree* - *Strongly agree* | 15 (8%)  18 (9%)  12 (6%)  49 (25%)  100 (52%) |

Abbreviations: *APOE* = apolipoprotein-E gene.

**Supplementary Table 6:** Factors influencing blood biomarker test selection

| **Survey questions** | **Response, n (%)** |
| --- | --- |
| Test method (i.e. immunoassay versus mass spectrometry)   - *Not at all important* - *Slightly important* - *Important* - *Fairly important* - *Very important* | 43 (24%)  30 (17%)  51 (28%)  31 (17%)  26 (14%) |
| Assay availability in the local laboratory   - *Not at all important* - *Slightly important* - *Important* - *Fairly important* - *Very important* | 13 (7%)  23 (13%)  45 (25%)  33 (18%)  67 (37%) |
| Analytical performance data are available for the test being ordered (e.g. precision, accuracy)   - *Not at all important* - *Slightly important* - *Important* - *Fairly important* - *Very important* | 5 (3%)  6 (3%)  25 (14%)  29 (16%)  116 (64%) |
| Clinical performance data are available for the test being ordered and the intended use population (e.g. sensitivity and specificity in comparison to gold standard biomarkers)   - *Not at all important* - *Slightly important* - *Important* - *Fairly important* - *Very important* | 0 (0%)  3 (2%)  23 (13%)  24 (13%)  131 (72%) |
| Test has been validated on the population that would be using the test (e.g. clinical status, diversity)   - *Not at all important* - *Slightly important* - *Important* - *Fairly important* - *Very important* | 0 (0%)  2 (1%)  19 (10%)  28 (15%)  132 (73%) |
| Turn-around time of the results   - *Not at all important* - *Slightly important* - *Important* - *Fairly important* - *Very important* | 4 (2%)  31 (17%)  63 (35%)  60 (33%)  23 (13%) |
| Knowledge of how comorbidities (i.e. chronic kidney disease, obesity, other health conditions) might affect the interpretation of the test being ordered   - *Not at all important* - *Slightly important* - *Important* - *Fairly important* - *Very important* | 1 (11%)  2 (1%)  31 (17%)  36 (20%)  111 (61%) |
| Financial cost of the test (to the health care system or to the patient)   - *Not at all important* - *Slightly important* - *Important* - *Fairly important* - *Very important* | 2 (1%)  15 (8%)  49 (27%)  39 (22%)  76 (42%) |

Abbreviations: AD = Alzheimer’s disease

**Supplementary Table 7:** Considerations when reviewing biomarker test results

| **Survey questions** | **Response, n (%)** |
| --- | --- |
| Information about the assay imprecision or diagnostic uncertainty of results near the clinical decision limit cut-point   - *Not at all important* - *Slightly important* - *Important* - *Fairly important* - *Very important* | 2 (1%)  8 (5%)  36 (20%)  41 (23%)  90 (51%) |
| Assay methodology being used   - *Not at all important* - *Slightly important* - *Important* - *Fairly important* - *Very important* | 14 (8%)  46 (26%)  54 (31%)  34 (19%)  29 (16%) |
| Clinical sensitivity and specificity of the cut-point(s) used in the assay in specified contexts of use relevant for my setting   - *Not at all important* - *Slightly important* - *Important* - *Fairly important* - *Very important* | 3 (2%)  5 (3%)  24 (14%)  33 (19%)  112 (63%) |
| Positive and negative predictive value of the test)   - *Not at all important* - *Slightly important* - *Important* - *Fairly important* - *Very important* | 3 (2%)  6 (3%)  26 (15%)  42 (24%)  100 (56%) |
| Information on how to interpret a result in specific clinical contexts, including known comorbidities   - *Not at all important* - *Slightly important* - *Important* - *Fairly important* - *Very important* | 2 (1%)  4 (2%)  22 (12%)  28 (16%)  121 (68%) |

Abbreviations: AD = Alzheimer’s disease

**Supplementary Table 8:** Pre-test counseling and education for patients

| **Survey questions** | **Response, n (%)** |
| --- | --- |
| Numbers of false positive and false negative results   - *Not at all important* - *Slightly important* - *Important* - *Fairly important* - *Very important* | 4 (2%)  11 (6%)  28 (16%)  47 (27%)  83 (48%) |
| Factors that may affect the test results aside from the presence of AD pathology (demographics, comorbidities)   - *Not at all important* - *Slightly important* - *Important* - *Fairly important* - *Very important* | 3 (2%)  6 (3%)  33 (19%)  45 (26%)  86 (50%) |
| Assay performance in patient's ethnic group and geography   - *Not at all important* - *Slightly important* - *Important* - *Fairly important* - *Very important* | 5 (3%)  16 (9%)  32 (18%)  36 (21%)  84 (49%) |
| Implications of the result for treatment decisions   - *Not at all important* - *Slightly important* - *Important* - *Fairly important* - *Very important* | 2 (1%)  3 (2%)  20 (12%)  33 (19%)  115 (66%) |
| How an indeterminate result will be handled/interpreted   - *Not at all important* - *Slightly important* - *Important* - *Fairly important* - *Very important* | 4 (2%)  3 (2%)  24 (14%)  39 (23%)  103 (60%) |
| Situations in which there will be a need for confirmatory tests (CSF/amyloid PET)   - *Not at all important* - *Slightly important* - *Important* - *Fairly important* - *Very important* | 2 (1%)  3 (2%)  20 (12%)  42 (24%)  106 (61%) |

Abbreviations: AD = Alzheimer’s disease; CSF = cerebrospinal fluid; PET = positron emission tomography.
